# Determinants of birth asphyxia among newborn live births in public hospitals of Gamo and Gofa zones, Southern Ethiopia

**DOI:** 10.1186/s12887-022-03342-x

**Published:** 2022-05-13

**Authors:** Kebebew Lemma, Direslgne Misker, Mekidim Kassa, Hanan Abdulkadir, Kusse Otayto

**Affiliations:** 1 Shashemene Comprehensive Specialized Hospital, Shashemene, Ethiopia; 2 School of Public Health, College of Medicine and Health Sciences, Arba Minch University, Arba Minch, Ethiopia

**Keywords:** Birth asphyxia, Newborn, Neonates, Perinatal, Case–control

## Abstract

**Background:**

Birth asphyxia is the inability of a newborn to start and conserve breathing immediately after birth. Globally, 2.5 million infants die within their first month of life every year, contributing nearly 47% of all deaths of children. It is the third cause of neonatal deaths next to infections and preterm birth. Ethiopia is one of the countries with the highest neonatal mortality and high burden of birth asphyxia in the world. The state of birth asphyxia is about 22.52% in Ethiopia, with incidence of 18.0% in East Africa Neonatal mortality incidence ratio was 9.6 deaths per 1000 live births among which 13.5% of neonatal mortality cases were due to birth asphyxia in southern Ethiopia. The effect of birth asphyxia is not only limited to common clinical problems and death; it also has a socio-economic impact on the families. Therefore, this study is aimed to identify determinants of birth asphyxia among newborn live births in public hospitals Southern Ethiopia.

**Methods:**

An Institution based unmatched case–control study was conducted among newborn live births in public hospitals of Gamo & Gofa zones, with a total sample size of 356 (89 cases and 267 controls, 1:3 case to control ratio) from March 18 to June 18, 2021, after obtaining ethical clearance from Arba Minch University. Cases were selected consecutively and controls were selected by systematic random sampling method. Data were collected using an adapted pretested semi-structured questionnaire through face-to-face interviews and record reviews using an extraction checklist for intrapartum & neonatal-related information. The collected data were entered into Epi data version 4.4 and exported to STATA version 14 for analysis. Finally, bi-variable and multivariable logistic regression analyses were performed to identify determinants of birth asphyxia. Statistical significance was declared at *p*-value ≤ 0.05 along with corresponding 95% CI of AOR used to declare statistical significance. Results: Anemia during pregnancy [AOR = 3.87, 95% CI (1.06- 14.09)], breech presentation [AOR = 3.56, 95% CI (1.19–10.65)], meconium stained amniotic fluid [AOR = 6.16, 95% CI (1.95–19.46)], cord prolapse [AOR = 4.69, 95%CI (1.04–21.05)], intrapartum fetal distress [AOR = 9.83, 95% CI (3.82–25.25)] and instrumental delivery [AOR = 5.91, 95% CI (1.51–23.07)] were significantly associated with birth asphyxia.

**Conclusion:**

The study revealed that anemia during pregnancy, breech presentation, meconium-stained amniotic fluid, cord prolapse, intrapartum fetal distress, and instrumental delivery were identified as determinants of birth asphyxia. Therefore, health professional and health institutions should give emphasis on care of mother and the newborn in actively detecting and managing asphyxia.

**Supplementary Information:**

The online version contains supplementary material available at 10.1186/s12887-022-03342-x.

## Background

Birth asphyxia is the inability of a newborn to start and conserve breathing immediately after birth [1]. It is the default of the newborn to sustain adequate respiration just after delivery [2]. It happens when the brain and other organs do not get adequate oxygen & nutrients. It can occur before, during, or after birth [3].

According to International Classification of Disease (ICD 11) birth asphyxia is diagnosed as asphyxia when the APGAR (Appearance/Color, Pulse/Heart rate, Grimaces/Reflexes, Activity/Muscle tone, and Respiration) score at 5^th^ minute is less than seven by the two levels, which is given a score of 0, 1, or 2 accordingly [2, 4].

Every year, about 2.5 million infants die within the first twenty-eight days of their age globally, this kick in nearly 47% of all deaths of under-five. Reports indicated that birth asphyxia is the third cause of neonatal deaths next to infections and preterm birth [5]. Nearly 23% of the neonatal deaths and 29% of early neonatal deaths around the world are accredited to birth asphyxia yearly [6]. Almost all deaths of newborns are in developing countries, with the highest number in South Asia and sub-Saharan Africa (SSA) [5]. About 25% of the world's newborn deaths have occurred in Africa, of those, birth asphyxia accounts for 24% [7].

Ethiopia is one of the countries with the highest neonatal mortality in the world. There is high burden of birth asphyxia in Ethiopia. An umbrella review of state of birth asphyxia revealed about 22.52% asphyxia occurs in Ethiopia with incidence of 18.0% in East Africa [8]. Ethiopian mini Demographic Health Surveillance (EDHS), 2019 report also indicated that there is a slight increase in neonatal mortality rate which was 29 deaths per 1000 live births in the 2016 EDHS report & 30 in 2019 [9, 10]. In the southern Ethiopia, Gamo & Gofa zones neonatal mortality incidence ratio was 9.6 deaths per 1000 live births among which 13.5% of neonatal mortality cases were due to birth asphyxia in 2019 [11].

Serious neonatal complications noted among the asphyxiated babies were hypoxic-ischemic encephalopathy (HIE) with convulsion, neonatal jaundice, septicemia, transient tachypnea of neonate, hypoglycemia, respiratory distress syndrome, caput succedaneum, and feeding problem [12]. Asphyxia is significantly associated with an increased risk of impaired renal function in preterm neonates within the first day after birth [13]. Asphyxia without intervention results in ongoing circulatory deterioration eventually leading to myocardial dysfunction, circulatory shock, right and left ventricular failure, tricuspid regurgitation, hypotension, and eventually cardiac arrest [14].

The effect of birth asphyxia is not limited only to common clinical problems & death it also has an impact on a subsequent socioeconomic burden on the families. According to findings from Accra of Ghana, families of asphyxiated neonates spent high medical-related costs & out-of-pocket payments irrespective of health insurance status. On average, families spent are 9.1% of their annual income on acute look after perinatal asphyxia [15].

Factors for birth asphyxia can be divided into antepartum, intrapartum, and fetal [16]. It is a common and serious neonatal problem that upshot neonatal morbidity and mortality. In developing countries majority of the cases suffer from the consequences of birth asphyxia. In Ethiopia, birth asphyxia is a major cause of neonatal mortality and morbidity.

The information regarding birth asphyxia is limited in the study area. So the study aims to spot determinants of birth asphyxia among newborn live births delivered in public hospitals of Gamo and Gofa zones, Southern Ethiopia.

## Methods and materials

### Study area, study design and study period

An institution based unmatched case control study was conducted in public hospitals of Gamo & Gofa zones, southern Ethiopia. Gamo and Gofa are zones within South Nation, Nationalities, and Peoples Region (SNNPR). Arba Minch town is the administrative center of the Gamo zone, which is located 505 km away from Addis Ababa the capital city of Ethiopia. The total population of the Gamo zone is 1,580,042 (790,372 males and 789,670 female) among which under five and reproductive age groups accounts for 15.6% and 23.3% of the total population respectively. The zone is divided into 18 administrative Woreda and four towns administrate. There are five public hospitals, 56 health centers, and 302 health posts in the Gamo zone. Gofa zone is located 516 km away from the capital, Addis Ababa. The zone contains 7 rural administrative Woreda & two city administrations. The total population of Gofa zone is 658005 (M = 326,868; *F* = 333,472). There are 25 health centers, 178 health posts, one general, and one district hospitals [17]. The study was conducted from March 18 to June 18, 2021.

### Population

All newborn live births delivered at public hospitals of Gamo and Gofa zones were the source population whereas newborn live babies who were born at the public hospitals of Gamo and Gofa zones during the data collection period were the study population for this study.

The Cases and Controls were identified from the medical records of the mother.

**For cases:** All asphyxiated newborns with APGAR scores of less than 7 at the 5^th^ minute, delivered after 28 weeks of gestation and confirmed by the physician in public hospitals of Gamo and Gofa zones during the study period.

**For controls:** Newborns delivered after viability and not diagnosed with birth asphyxia (without asphyxia) who cry, breath without difficulty, not gasping, APGAR scores ≥ 7 at 5^th^ minute and selected systematically in public hospitals of Gamo and Gofa zones during the study period.

Newborn live neonates who were born either by SVD, IVD, or CS in public hospitals of Gamo and Gofa zones during the study period were included in this study. However, neonates born with congenital malformations and data with incomplete documentation was excluded from the study.

### Sample size determination

The sample size is calculated by using Epi Info version 7.0 software sample size calculation program for an unmatched case–control study. Considering the following assumptions: 5% level of significance, power of 80%, control to case ratio of 3:1 and the different exposure variables like birth weight < 2500gm, instrumental delivery, and gestational age in weeks > 42 from previous studies [18–20]. Finally, by adding 10% non-response rate the total sample size was 356 (89 cases and 267 controls).

### Sampling technique

All the Public hospitals in Gamo and Gofa zones were selected purposely because of the small number of cases and give a total of 7 Hospitals (5 from the Gamo zone and two from the Gofa zone). The total sample size was proportionally allocated to each hospital with their respective previous two-month delivery report.

Subjects are divided into cases and controls. All neonates who delivered with an APGAR score of less than seven at the fifth minute and confirmed by physicians were taken as a case until the required sample size was fulfilled (Consecutive sampling technique). When a single case of birth asphyxia was observed in any of the seven hospitals the data collectors immediately investigated a comparable control longitudinally. Controls were selected using systematic random sampling technique by getting the K value which is obtained by dividing the total number of non-asphyxiated newborns from each hospital to the required number of controls for the study in each hospital.

$$\mathrm{K}= \frac{\mathrm{Total number of controls from each hospitals monthly report}}{\mathrm{The required number of controls in each hospital}}= 6$$

Proportional allocation of respondents to the seven hospitals was done. A total of 356 (89 cases and 267 controls, 1:3 case to control ratio) participants were included in the study.

### Operational definition and definition of terms

**Birth asphyxia:** is diagnosed when a newborn with any of the signs of impaired breathing (not breathing or not crying, gasping, cyanosis, and < 30 breaths per minute) at birth with an APGAR score less than 7 at 5^th^ minutes and confirmed by the physician [4, 21].

**Partograph used:** The documentation status of the parameters was defined based on the time interval of documentation. Descent of the fetal head, uterine contractions, maternal blood pressure, respiration, and pulse rate should be monitored every hour, molding & cervical dilation every 4 h, temperature every 2 h, and fetal heart rate every 30 min. If all the criteria are satisfied for each parameter on the partograph, the partograph is considered fully done.

**Partograph not used:** Each parameter recorded on partograph not meeting any of the accepted time intervals or with parts misplaced/missing, if no information was documented on the parameters of partograph or if the partograph sheet is not inserted on medical records of mother was regarded as not used.

**Incomplete data:** Documents/Medical records that contain any missed parameters of outcome variable and documents that miss > 20% of independent variables (intrapartum and neonatal-related information) was considered as incomplete.

**Meconium stained amniotic fluid (MSAF):** if the amniotic fluid was green/brown or mixed with meconium, or appears meconium-stained on the baby.

**Prolonged labor:** when the labor, after the latent phase of the first stage of labor, exceeds 12 h in prim gravida or 8 h in multipara mothers.

**Premature rupture of membranes (PROM):** if a rupture of the membrane of the amniotic sac and chorion occurs for more than one hour before the onset of labor.

**Preeclampsia/eclampsia:** is a during pregnancy complication characterized by high blood pressure and protein in the urine. If not managed, preeclampsia can progress to eclampsia, which is defined as the development of seizures in a woman with preeclampsia.

**Gaya Smoking:** is a form of local tobacco smoking engaged by men and women by using a pipe.

**Anemia:** In pregnant women defined when the hematocrit level is < 33%.

**Oligohydramnios:** When the volume of amniotic fluid is deficient: less than 500 ml.

**Polyhydramnios:** An excessive amount of amniotic fluid usually exceeding 2L.

### Data collection instrument and procedure

Maternal socio-demographic and antepartum related data were collected using semi-structured interviewer-based questionnaire. Data on intrapartum and neonatal related factors were abstracted using a structured checklist from the medical records of mothers who gave birth during the study period.

Neonatal birth asphyxia was determined using APGAR score. Those newborn babies with APGAR score of < 7 at 5^th^ minutes & diagnosed with asphyxia by the physician were taken as cases while those with a score of greater than/equal to seven were taken as controls. The data was collected by seven Female nurses working in NICU and supervised by seven senior Nurses. For the face-to-face interview technique, proper orientation was given for each participant on the purpose and usefulness of the study and after getting written consent, face-to-face interviewing respondents was cascaded based on questionnaires.

### Data quality control

To assure the data quality, a data collection tool was prepared after an intensive review of relevant literature and similar studies. Properly designed data collection instruments were provided after translation into Amharic language and retranslated back to English by a language expert to check for inconsistency. The English version of the checklist was used to retrieve data from the mothers’ medical records. Appropriate training was given for data collectors and supervisors. The training was including a briefing on the purpose of the study, approach of accessing study participants, clarity on each item in the instruments, data collecting procedure, inclusion or exclusion of the target data source, timeliness of data submission, data handling, and time management. Pre-testing was performed at Otona General Hospital (OGH) on 5% of the sample size one week before the actual data collection and the necessary correction was made based on the pretest result to avoid confusion any and for better completion of the questions. Every day the collected data was reviewed and cross-checked for completeness by the supervisors and weekly by the investigator.

### Data processing and analysis

The collected data were coded, cleaned, and entered by Epi-data version 4.4 and exported into STATA version 14 for analysis. Descriptive analysis was carried out and summarized by frequency tables, graphs, and text. Frequency and cross-tabulations were used to check for missing values.

A logistic regression model was used for both bivariate and multivariate analysis to identify determinants of birth asphyxia among groups of independent variables. Independent variables with a *p*-value of < 0.25, biologically plausible & showed significant association in the previous studies were included in the multivariable analysis to control for all possible confounders. The Hosmer & Lemeshow statistics were used to check the goodness of fit of the model. Variance inflation factor (VIF) was used to assess multicollinearity. However, no multicollinearity was detected as the variance inflation factor was < 5. Adjusted odds ratio (AOR) with 95% CI, was estimated to assess the strength of associations, and statistical significance was declared at a *p*-value ≤ 0.05. Results were presented using tables, figures, and texts.

## Results

### Descriptive statistics results

#### Socio-demographic characteristics

A total of 356 (89 cases and 267 controls) live births delivered at public hospitals of Gamo and Gofa zones were included with their mother making a response rate of 100%. Regarding the maternal age majority of mothers of the case and control groups were in the age category of 20 to 34 years, which was 73 (82.02%) and 223 (83.52%) respectively. The maternal age category beyond 35 years’ accounts for 12 (13.48%) and 30 (11.24%) for cases and controls respectively. The mean age of the mothers with cases and controls were 27.6 (SD ± 5.4) and 27.8 (SD ± 5.06) years respectively.

About concerning the residence of respondents, the majority residence among cases, 47 (52.8%) was rural dwellers while 160 (59.9%) of controls were from urban. Concerning the occupation of the mothers 61 (68.5%) of cases and 147 (55%) of controls were housewives (Table 1).

**Table 1:** Socio-demographic characteristics of the mothers for the determinants of birth asphyxia among newborn live births in public hospitals of Gamo and Gofa zones, Southern Ethiopia, 2021

Variables	Category	Case(*n* = 89)	Control(*n* = 267)	Total(*n* = 356)
		**n (%)**	**n(%)**	**n(%)(%)**
**Age of Mother**	15–19	4 (4.49)	14 (5.24)	18 (5.06)
	20–34	73 (82.02)	223 (83.52)	296 (83.15)
	35 +	12 (13.48)	30 (11.24)	42 (11.80)
**Marital status**	Married	84(94.38)	261(97.75)	345 (96.61)
	Single	5(5.62)	6(2.25)	11(3.09)
**Religion**	Orthodox	48 (53.93)	123 (46.07)	171 (48.03)
	Muslim	4 (4.49)	30 (11.24)	34 (9.55)
	Protestant	37 (41.37)	114 (42.70)	151 (42.42)
**Ethnicity**	Gamo	62 (69.66)	138 (51.69)	200 (56.18)
	Gofa	18 (20.22)	79 (29.59)	97(27.25)
	Wolaita	4(4.49)	13(4.87)	17(4.78)
	Amara	3(3.37)	18(6.74)	21(5.90)
	Other	2(2.25)	19(7.120	21(5.90)
**Residency**	Rural	47(52.81)	107(40.07)	154(43.26)
	Urban	42(47.19)	160(59.93)	202(56.74)
**Occupation**	Housewife	61(68.54)	147(55.06)	208(58.43)
	Governmental Employee	16(17.98)	65(24.34)	81(22.75)
	Merchant	9(10.11)	47(17.60)	56(15.73)
	Daily Labor	3(3.37)	8(3.00)	11(3.09)
**Gravidity**	Primigravida	31(34.83)	48(17.98)	79(22.19)
	Multigravida	58(65.17)	219(82.02)	277(77.81)
**Parity**	Primiparous	34(38.20)	54(20.22)	88(24.72)
	Multiparous	55(61.80)	213(79.78)	268(75.28)
**Birth spacing (in years)**	< = 2	35 (60.34)	132 (60.27)	167 (60.29)
	2	23 (39.66)	87 (39.73)	110 (39.71)
**MUAC in cm**	< 23	31(34.83)	77(28.84)	108(30.34)
	> = 23	58(65.17)	190(71.16)	248(69.66)
**History of adverse pregnancy outcome**	Yes	9(10.11)	22(8.24)	31(8.71)
	No	80(89.89)	245(91.76)	325(91.29)
**IUFD**	Yes	2 (2.25)	9(3.37)	11 (3.09)
	No	87 (97.75)	260 (97.38)	345 (96.91)
**Preterm**	Yes	4(4.49)	9(3.37)	13(3.65)
	No	85(95.51)	258(96.63)	343 (96.35)
**Neonatal death**	Yes	3(3.37)	8 (3.00)	11(3.09)
	No	86(96.63)	259 (97.00)	345 (96.91)

**Fig. 1:**
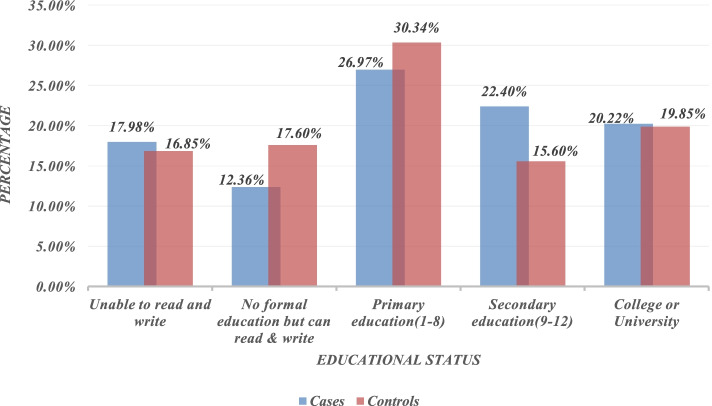
Levels of education among cases and controls for the determinants of birth asphyxia among newborn live births in public hospitals of Gamo and Gofa zones, southern Ethiopia, 2021

Newborns delivered from mothers with primary education levels were higher in a proportion of birth asphyxia in both cases (26.97%) and controls (30.37%) compared to other educational levels (Fig. 1).

#### Antepartum related characteristics

The majority of mothers, 78 (87.64%) of cases and 252 (94.38) of controls had received ANC follow-up while 11 (12.36%) of cases and 15 (5.62%) of controls had never got ANC follow-up during their pregnancy time of the current neonate.

The proportion of mothers, those visit less than four ANC visits was 48 (61.58%) in cases and 183 (72.62%) in controls. Among study participants, 35 (39.3%) of the mothers with cases and 71 (26.5%) of the mothers with control had a history of medical illness during their pregnancy period. Two (2.25%) of mothers with cases and 5 (1.87%) of mothers with controls had chronic Hypertension (Table 2).

**Table 2:** Antepartum characteristics of the mothers for the determinants of birth asphyxia among newborn live births in public hospitals of Gamo and Gofa zones, Southern Ethiopia, 2021

	**Variables**	**Case(*n* = 89)**	**Control(*n* = 267)**	**Total(*n* = 356)**
		n (%)	n (%)	n (%)
**ANC Follow up**	Yes	78(87.64)	252(94.38)	330(92.70)
	No	11(12.36)	15(5.62)	26(7.30)
**No of ANC visits**	1–3	48(61.54)	183(72.62)	231(70.00)
	> = 4	30( 38.46)	69(27.38)	99(30.00)
**Place of ANC visits**	Governmental HC	46(58.97)	144(57.14)	190(57.58)
	Governmental Hospital	32(41.03)	108(42.86)	140(42.42)
**Medical illness during Pregnancy**	Yes	35(39.33)	71(26.59)	106(29.78)
	No	54(60.67)	196(73.41)	250(70.22)
**Pregnancy induced HTN**	Yes	11(12.36)	10(3.75)	21(5.90)
	No	78(87.64)	257(96.25)	335(94.10)
**APH**	Yes	8 (8.99)	9 (3.37)	17 (4.78)
	No	81 (91.01)	258 (96.63)	339 (95.22)
**Anemia**	Yes	16(17.97)	18 (6.74)	34(9.55)
	No	73 (82.02)	249 (93.26)	322(90.45)
**Infections**	Yes	4 (4.49)	8 (3.00)	12 (3.37)
	No	85 (95.51)	259 (97.00)	344 (96.63)
**Gestational diabetes**	Yes	2 (2.25)	6 (2.25)	8 (2.25)
	No	87 ( 97.75)	261 (97.75)	348 (97.75)
**DM**	Yes	3 (3.37)	5 (1.87)	8 (2.25)
	No	86 (96.63)	262 (98.13)	348 (97.75)
**HTN**	Yes	2(2.25)	5 (1.87)	7 (1.97)
	No	87 (97.75)	262 (98.13)	349 (98.03)
**Pre-eclampsia/eclampsia**	Yes	6 (6.74)	8 (3.00)	14 (3.93)
	No	83 (93.26)	259 (97.00)	342 (96.07)
**Malaria**	Yes	8 (8.99)	22 (8.24)	30 (8.43)
	No	81 (91.01)	245 (91.76)	326 ( 91.57)
**Syphilis**	Yes	1 (1.12)	4 (1.50)	5 (1.40)
	No	88 (98.88)	263 (98.50)	351( 98.60)
**Other illnesses**	Yes	3 (3.37)	6 (2.25)	9 (2.53)
	No	86 (96.63)	261 ( 97.75)	347 (97.47)
**Substance use during Pregnancy**	Yes	12 (13.48)	31 (11.61)	43 (12.08)
	No	77 (86.52)	236 (88.39)	313 (87.92)
**Abortion**	Yes	7 (7.87)	13 (4.87)	20 (5.62)
	No	82 (92.13)	254 (95.13)	336 (94.38)
**No of Abortion**	One Abortion	6 (85.71)	10 (76.92)	16 (80.00)
	> 1 abortion	1 (14.29)	3 (23.08)	4(20.00)

**Fig. 2:**
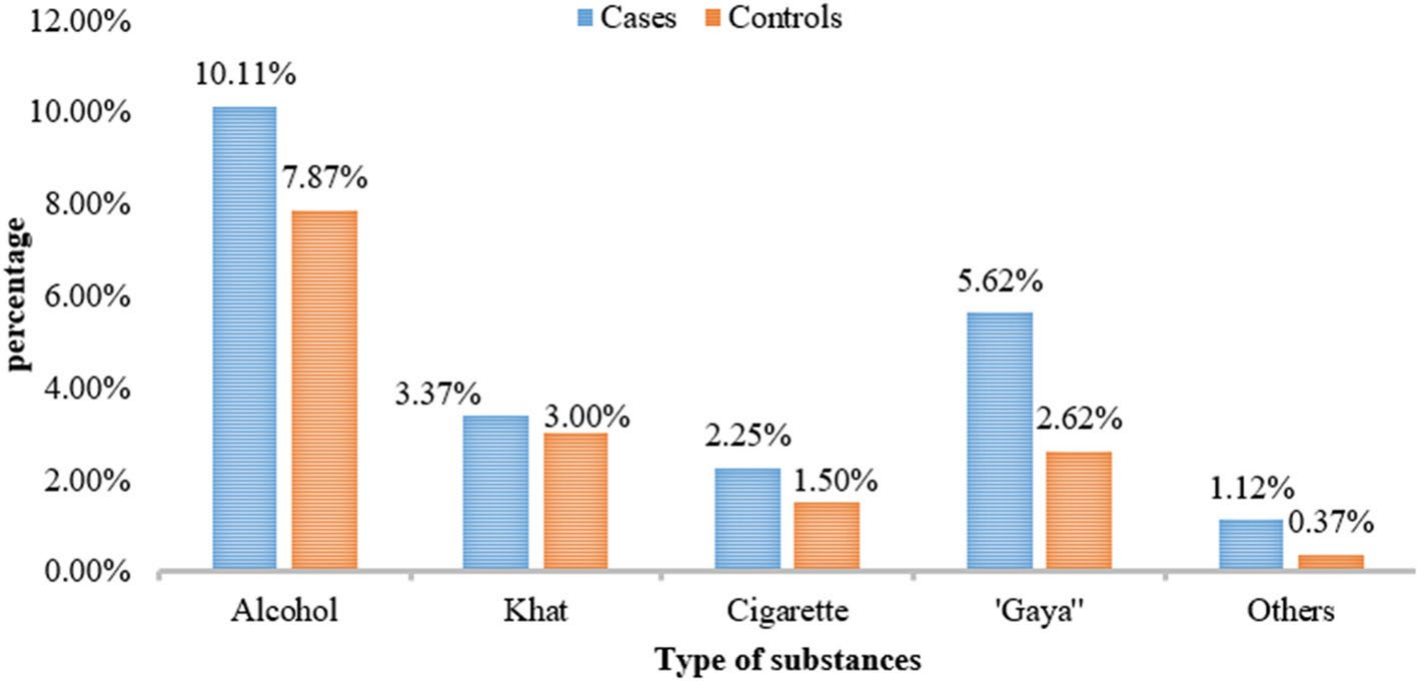
History of substance use during pregnancy among mothers of neonates with cases and controls for the determinants of birth asphyxia among newborn live births in public hospitals of Gamo and Gofa zones, southern Ethiopia, 2021

Concerning the history of substance use during pregnancy 5.62% of cases and 2.62% of controls used to smoke “Gaya” (Fig. 2).

#### Intrapartum related factors

Thirty-one (34.83%) of cases and 213 (79.78%) control mothers were delivered spontaneously and 57 (64.04%) cases and 245(91.76%) control mothers were having vertex presentation while 32(35.96%) cases and 22(8.24%) controls had breech presentations. Forty-eight (53.9%) cases and 27 (10.11%) control mothers were having an experience of prolonged labor and 33 (37.08%) cases and 25 (9.36%) control mothers were having obstructed labor as a complication of labor. Among the mothers of infants, 25 (28.09%) of cases and 29 (10.86%) of controls faced premature rupture of membrane before labor starts. Only 62 (69.66%) of cases and 226 (84.64%) of controls were followed by partographs during labor. Majority of the cases 48 (53.93%) were delivered at night time while the majority of controls 182 (68.16%) were delivered at day time.

The proportion of prolonged labor was 48(53.9%) in cases and 27(10%) among controls. Forty-seven (52.81%) cases and 13(4.87%) of control mothers had meconium stained on pelvic examination (Table 3).

**Table 3:** Antepartum characteristics of the mothers for the determinants of birth asphyxia among newborn live births in public hospitals of Gamo and Gofa zones, Southern Ethiopia, 2021

Variables	Category	Case(*n* = 89)	Control(*n* = 267)	Total(*n* = 356)
		n (%)	n (%)	n (%)
**Partograph used**	Yes	62 (69.66)	226 (84.64)	288 (80.90)
	No	27 ( 30.34)	41 ( 15.36)	68 (19.10)
**Presentation**	Vertex	57 (64.04)	245 (91.76)	302 (84.83)
	Breech	32 ( 35.96)	22(8.24)	48 ( 15.17)
**Labor Type**	Spontaneous	72(80.90)	250 (93.63)	322 (90.45)
	Induced	17 ( 19.10)	17 ( 6.37)	34( 9.55)
**Time of Membrane rapture**	PROM	25 (28.09)	29 (10.86)	54 (15.17)
	Intrapartum	64 (71.91)	238 (89.14)	302 (84.83)
**Maternal Fever**	Yes	22 (24.72)	21 (7.87)	43 (12.08)
	No	67 (75.28)	246 ( 92.13)	313 (87.92)
**Labor duration**	Normal	41 (46.07)	240 (89.89)	281 (78.93)
	Prolonged	48 ( 53.93)	27 (10.11)	75 (21.07)
**Color of amniotic fluid**	Meconium stained	47 (52.81)	13 (4.87)	60 (16.85)
	Clear	42 (47.19)	254 (95.13)	296 (83.15)
**Delivery time**	Night	48 (53.93)	85 (31.84)	133 (37.36)
	Day	41 (46.07)	182 ( 68.16)	223 (62.64)
**Mode of Delivery**	SVD	31 (34.83)	213 (79.78)	244 (68.54)
	C/S	40 (44.94)	44 (16.48)	84(23.60)
	Instrumental Delivery	18 (20.22)	10 (3.75)	28 (7.87)
**Type of anesthesia**	General anesthesia	4 (10)	5 (11.36)	9 (10.71)
	Spinal anesthesia	36 (90)	39 (88.64)	75 (89.29)
**Birth attendant**	Doctor	15 (16.85)	20 (7.49)	36 (9.83)
	Midwife	42 (47.19)	221 (82.77)	263 (73.88)
	IESO	32 (35.96)	26 (9.74)	58 (16.29)
**Obstructed labor**	Yes	33 (37.08)	25 (9.36)	58 (16.29)
	No	56 (62.92)	242 (90.64)	298 (83.71)
**Maternal Hypotension**	Yes	4 (4.49)	7 (2.62)	11 (3.09)
	No	85 (95.51)	260 (97.38)	345 (96.91)
**Placenta abruption**	Yes	3 (3.37)	6 (2.25)	9 (2.53)
	No	86 (96.63)	261 (97.75)	347 (97.47)
**Placenta Previa**	Yes	8 (8.99)	5 (1.87)	13 (3.65)
	No	81 (91.01)	262 (98.13)	343 (96.35)
**FHR**	< 100, > 180	70 (78.65)	38 (14.23)	108 (30.34)
	100–180	19 (21.35)	229 (85.77)	248 (69.66)
**Oligohydramnios**	Yes	3 (3.37)	5 (1.87)	8 (2.25)
	No	86 (96.63)	262 (98.13)	348 (97.75)
**Polyhydramnios**	Yes	2 (2.25)	4 (1.50)	6 (1.69)
	No	87 (97.75)	263 (98.50)	350 (98.31)
**Cord Prolapse**	Yes	9 (10.11)	9 (3.37)	18 (5.06)
	No	80 (89.89)	258 (96.63)	338 (94.94)

#### Neonatal related factors

The majority of cases, 56 (62.92%) and 162 (60.67%) of control were male neonates. Among all sexes, the proportion of low birth weight was 15 (16.85%) of cases and 27 (10.11%) of controls. Preterm babies contributed 21 (23.60%) of cases and 38 (14.23%) of controls. In cross-tabulation with birth weight, preterm babies contributed 27 (8%) of low birth weight (Table 4).

**Table 4:** Neonatal related characteristics of the mothers for the determinants of birth asphyxia among newborn livebirths in public hospitals of Gamo and Gofa zones, Southern Ethiopia, 2021

Variables	Category	Case(*n* = 89)	Control(*n* = 267)	Total(*n* = 356)
		n (%)	n (%)	n (%)
**Sex of neonate**	Male	56 (62.92)	162(60.67)	218 (61.24)
	Female	33(37.08)	105 (39.33)	138 (38.76)
**Birth type**	Singleton	86 (96.63)	258 (96.63)	344 (96.63)
	Twin	3 (3.37)	9 (3.37)	12 (3.37)
**Gestational age at birth (in weeks)**	< 37	21 (23.60)	38 (14.23)	59 (16.57)
	37- 42	62 (69.66)	218 (81.65)	280 (78.65)
	> = 42	6 (6.74)	11 (4.12)	17 (4.78)
**Birth weight (in grams)**	< 2500	15 (16.85)	27 (10.11)	42 (11.80)
	2500–4000	65 (73.03)	226 (84.64)	291 (81.74)
	> 4000	9 (10.11)	14 (5.24)	23 (6.46)

#### Determinants of birth asphyxia

A bivariate logistic regression was run to identify candidate variables for multivariable regression analysis. In bivariate regression analysis variables: residence, gravidity, parity, number of ANC visits, pre-eclampsia/eclampsia, anemia during pregnancy, APH, pregnancy-induced hypertension, history of ‘Gaya’ smoking during pregnancy, delivery not followed by partograph, fetal presentation, mode of delivery, obstructed labor, labor duration, delivery time, maternal fever, fetal distress, color of amniotic fluid, profession of birth attendant, gestational age at birth, cord prolapse, placenta Previa, time of rapture of membrane and birth weight were variables found to be statistically significant at *p*-value less than 0.25 and candidate for multivariate regression.

According to the bivariate regression findings of this study neonates born to primiparous mothers had about three times higher odds to develop asphyxia as compared to multiparous mothers.

Being a rural dweller had about twofold higher odds of birth asphyxia compared to urban dweller women.

The odds of perinatal asphyxia were nearly three times higher among those who developed preeclampsia or eclampsia than those who did not develop preeclampsia or eclampsia.

Regarding birth weight, the likelihood of asphyxia was two times higher among low birth weight neonates than the counterparts.

Newborns born from ‘Gaya’ smoker mothers had about two-fold higher odds of birth asphyxia compared to non-smokers (Table 5).

**Table 5:** Bivariate and multivariable analysis of determinants of birth asphyxia among newborn live births in public hospitals of Gamo and Gofa zones, Southern Ethiopia, 2021

Variables	Category	Case (*n* = 89)	Control (*n* = 267)	COR(95%CI)		AOR(95%CI)	*P*-value
		**n (%)**	**n (%)**				
**Residency**	Rural	47(52.81)	107(40.07)	1.67(1.03–2.71)		1.39(0.62–3.09)	0.417
	Urban	42(47.19)	160(59.93)	1		1	
**Gravidity**	Primigravida	31(34.83)	48(17.98)	2.43(1.42–4.16)		1.06(0.12–9.01)	0.957
	Multigravida	58(65.17)	219(82.02)	1		1	
**Parity**	Primiparous	34(38.20)	54(20.22)	2.43(1.44–4.10)		3.82(0.47–30.69)	0.207
	Multiparous	55(61.80)	213(79.78)	1		1	
**Antepartum related factors**							
**No of ANC visits**	1–3	48(61.54)	183(72.62)	0.60(0.35–1.02)	0.84(0.35–1.97)		0.694
	> = 4	30( 38.46)	69(27.38)	1	1		
**Pregnancy induced HTN**	Yes	11(12.36)	10(3.75)	3.62(1.48–8.85)	0.80(0.16–4.02)		0.791
	No	78(87.64)	257(96.25)	1	1		
**APH**	Yes	8 (8.99)	9 (3.37)	2.83(1.05–7.57)	1.24(0.17–8.78)		0.827
	No	81 (91.01)	258 (96.63)	1	1		
**Anemia**	Yes	16(17.97	18 (6.74)	3.03(1.47–6.24)	3.87(1.06–14.09)		0.040*
	No	73 (82.02)	249 (93.26)	1	1		
**Preeclampsia/eclampsia**	Yes	6 (6.74)	8 (3.00)	2.34 (0.78–6.93)	2.95 (0.60–14.34)		0.180
	No	83 (93.26)	259 (97.00)	1	1		
**‘Gaya smoking’**	Yes	5(5.62)	7(2.62)	2.21(0.68–7.14)	1.74(0.21–14.07)		0.600
	No	84(94.38)	260(97.38)	1	1		
**Intrapartum related factors**							
**Partograph used**	Yes	62 (69.66)	226 (84.64)	1		1	
	No	27 (30.34)	41 ( 15.36)	2.40(1.36–4.20)		0.74(0.26–2.10)	0.583
**Presentation**	Vertex	57 (64.04)	245 (91.76)	1		1	
	Breech	32(35.96)	22(8.24)	6.25(3.38–11.55)		3.56(1.19–10.65)	0.023*
**Labor Type**	Spontaneous	72(80.90)	250 (93.63)	1		1	
	Induced	17 (19.10)	17 ( 6.37)	3.47 (1.68–7.14)		0.63(0.16–2.35)	0.495
**Time of Membrane rapture**	PROM	25 (28.09)	29 (10.86)	3.20(1.75–5.85)		0.42(0.12–1.45)	0.174
	Intrapartum	64 (71.91)	238 (89.14)	1		1	
**Maternal Fever**	Yes	22(24.72)	21(7.87)	3.84(1.99–7.41)		0.91(0.29–2.86)	0.882
	No	67(75.28)	246( 92.13)	1		1	
**Labor duration**	Normal	41(46.07)	240 (89.89)	1		1	
	Prolonged	48(53.93)	27 (10.11)	10.40(5.84–18.51)		2.93(0.98–8.70)	0.053
**Color of amniotic fluid**	MS	34 (38.20)	14 (5.24)	11.17(5.61–22.21)		6.16(1.95–19.46)	< 0.002**
	Clear	55 (61.80)	253 (94.76)	1		1	
**Delivery time**	Night	48 (53.93)	85 (31.84)	2.50(1.53–4.09)		2.03(0.91–4.50)	0.081
	Day	41 (46.07)	182 (68.16)	1		1	
**Mode of Delivery**	SVD	31 (34.83)	213 (79.78)	1		1	
	C/S	40 (44.94)	44 (16.48)	6.24 (3.53–11.04)		2.55(0.72–8.94)	0.142
	ID	18 (20.22)	10 (3.75)	12.36(5.23–29.22)		5.91(1.51–23.07)	0.010**
**Birth attendant**	Doctor	15 (16.85)	20 (7.49)	3.94(1.87–8.32)		0.50(0.09–2.72)	0.427
	Midwife	42 (47.19)	221 (82.77)	1		1	
	IESO	32 (35.96)	26 (9.74)	6.47(3.50–11.96)		0.95(0.27–3.30)	0.939
**Obstructed labor**	Yes	33 (37.08)	25 (9.36)	5.70(3.14–10.34)		0.33(0.10–1.07)	0.067
	No	56 (62.92)	242 (90.64)	1		1	
**FHR**	< 100 or > 180	70 (78.65)	38 (14.23)	22.20(12.03–40.95)		9.83(3.82–25.25)	< 0.001***
	100–180	19 (21.35)	229 (85.77)	1		1	
**Cord Prolapse**	Yes	9 (10.11)	9 (3.37)	3.22(1.23–8.40)		4.69(1.04–21.05)	0.043*
	No	80 (89.89)	258 (96.63)	1		1	
**Placenta Previa**	Yes	8 (8.99)	5 (1.87)	5.17(1.64–16.25)		1.68(0.26–10.50)	0.579
	No	81 (91.01)	262 (98.13)	1		1	
**Neonatal related factors**							
**Gestational age at birth (in weeks)**	< 37	21 (23.60)	38 (14.23)	1.94(1.06–3.55)		1.52(0.44–5.16)	0.502
	37- 42	62 (69.66)	218 (81.65)	1		1	
	> = 42	6 (6.74)	11 (4.12)	1.91(0.68–5.39)		0.41(0.07–2.25)	0.309
**Birth weight (in grams)**	< 2500	15 (16.85)	27 (10.11)	1.93 (0.97–3.84)		1.24(0.28–5.35)	0.770
	2500–4000	65 (73.03)	226 (84.64)	1		1	
	> 4000	9 (10.11)	14 (5.24)	2.23(0.92–5.39)		1.18(0.26–5.38)	0.824

*FHR* Fetal heart rate*, APH* Antepartum hemorrhage*, MS* Meconium stained

^*^ = *p* ≤ 0.05*,*

^**^ = *p* < 0.01*,*

^***^ = *p* < 0.001

A total of twenty-five variables were included in multivariable logistic regression. After running multivariate logistic regression analysis, anemia during pregnancy, breech presentation, meconium-stained amniotic fluid, instrumental delivery, cord prolapse, and intrapartum fetal distress were found to be a statistically significant association in multivariable analysis.

The odds of having asphyxiated neonate is four times higher for mothers with anemia during pregnancy than mothers who did not have anemia during pregnancy [AOR = 3.87, 95% CI (1.06- 14.09)].

Newborns with the breech presentations had nearly four times higher odds of birth asphyxia compared to neonates with vertex presentation [AOR = 3.56, 95% CI (1.19–10.65)].

The odds of having meconium-stained amniotic fluid was about six times higher in asphyxiated neonates than non-asphyxiated neonates [AOR = 6.16, 95% CI (1.95–19.46)].

Similarly, newborns with a history of cord prolapse had increased the risk of having birth asphyxia nearly five folds than those without a history of cord prolapse [AOR = 4.69, 95%CI (1.04–21.05)].

Newborns delivered through instrumental delivery had six times higher odds of birth asphyxia than those delivered through spontaneous vaginal delivery [AOR = 5.91, 95% CI (1.51–23.07)].

Neonates with intrapartum fetal distress had approximately ten times higher odds to have birth asphyxia when compared with neonates with normal fetal heart [AOR = 9.83, 95% CI (3.82–25.25)].

## Discussion

The study aimed to identify determinants of birth asphyxia among newborn live births in public hospitals of the Gamo and Gofa zones. In this study maternal history of anemia, instrumental delivery, intrapartum fetal distress, meconium-stained aspiration fluid, and cord prolapse were identified as independent determinants of birth asphyxia.

Neonates born from anemic mothers during the pregnancy period were nearly four times more likely to develop asphyxia compared with newborn neonates born from mothers who did not have anemia during pregnancy. This result is further confirmed by a study conducted in Dilla, Ethiopia [22], and Jerusalem [23]. The possible explanation for this can be typically due to iron deficiency or other issues related to the hemoglobin that transports oxygen and it results in decrement of the blood and oxygen supply to the infant and, in turn, lead to birth asphyxia.

Newborns with meconium-stained amniotic fluid were about six fold more likely to have birth asphyxia compared to those newborns with clear amniotic fluid. This finding is supported by studies obtained from Addis Ababa, Arsi, Amhara, Tigray regions of Ethiopia [19, 20, 24–26], Kenya [27], Ghana Accra [28], and India [29]. The possible explanation of this is the presence of meconium in the amniotic fluid may lead to aspiration of it into the lung. This can further lead to lung inflammation, obstruction, and limited lung movement. Then due to limited gas exchange that could eventually result in asphyxia [19].

Newborns with the breech presentations had nearly four-fold higher odds of birth asphyxia compared to neonates with vertex presentation. This result is supported by other studies from different parts of Ethiopia [20, 30–32], Karachi [16], Cameroon [33], and Nigeria[34]. The possible explanation for this might be due to the fact that the non-vertex presentation had an increased risk of umbilical cord prolapse, head entrapment, and birth injury which result in oxygen deprivation and this leads to hypoxia and finally birth asphyxia.

Newborns delivered via instrumental vaginal delivery had six times higher odds of developing birth asphyxia than those delivered through spontaneous vaginal delivery. This finding is further confirmed by different studies [19, 31, 35]. The possible explanation for this might be due to the reason that indications for the instrumental delivery were mostly indicated for prolonged obstructed labor and non-reassuring fetal heartbeat in which neonates do not get enough oxygen. It might also be since instrumental vaginal delivery may result in hemorrhagic cranial injuries such as cephalo-hematoma and hemorrhage, which might finally cause birth asphyxia.

Neonates with history of intrapartum fetal distress had approximately ten times higher odds of birth asphyxia when compared with neonates with normal fetal heart-beat. A result is consistent with previous studies obtained from Gonder, Ethiopia [36], Karachi [16], and Iraq [37]. The possible reason for this might be since fetal distress occurs when the fetus does not get adequate oxygen during pregnancy/labor so that this can further cause asphyxia [19].

This study also found that newborns with a history of cord prolapse had nearly five-fold higher odds of birth asphyxia compared to those without a history of cord prolapse. This finding is similar to previous study findings conducted in Wolaita Sodo [38]. The possible explanation for this is umbilical cord is a channel that allows blood flow and oxygen between the placenta and fetus when it prolapsed the bold flow and oxygen transfer are blocked and this leads to hypoxia.

### Strength of the study

APGAR score and physician confirmation were used to identify birth asphyxia.

### Limitations of the study

This study recognized the following limitations: Some variables were collected from records and were difficult to get a few of them, conducted at institution level only, was subjected to recalling bias, and selection biases might result because it is conducted by case–control study design.

## Conclusions

The study identified Anemia during pregnancy, instrumental delivery, intrapartum fetal distress, meconium-stained amniotic fluid, breech presentation, and cord prolapse as determinants of birth asphyxia. Thus, health professional and health institutions should give emphasis on care of mother and the newborn in actively detecting and managing birth asphyxia.

## Supplementary Information

**Additional file 1.** 

## Data Availability

The datasets generated and/or analyzed during the current study are not publicly available due to confidentiality but are available from the corresponding author on reasonable request.
