# Supplementary material for: Determinants of birth asphyxia among newborn live births in public hospitals of Gamo and Gofa zones, Southern Ethiopia
Source: BMC Pediatr. 2022 May 13;22:280. doi: 10.1186/s12887-022-03342-x (PMC9099035; doi:10.1186/s12887-022-03342-x)
Supplement: Supplementary file 1 — Additional file 1. [file 12887_2022_3342_MOESM1_ESM.docx]

Annex I: Interviewer Administered Semi-structured Questionnaire English version.

***Arba Minch University, College of Medicine & Health Sciences, School of Public Health.***

An interviewer-administered a semi-structured Questionnaire developed to determine Socio-demographic and Antenatal determinants of birth asphyxia among newborn live births in public hospitals of Gamo & Gofa zones.

1. ***Questionnaire Code____________________***

2. Status:

- Case
- Control

3. Name of the hospital______________________

**General instruction**

1. For multiple-choice questions, choose the best possible answer/s

2. If your answer is not listed among alternatives, please tell your own answer/idea for the data collector.

Part I: Sociodemographic factors

| S/N | Factors | Response | Remark |
| --- | --- | --- | --- |
| 100 | Residence | 1. Rural 2. Urban |  |
| 101 | Age (years) | ________________ |  |
| 102 | Marital status | 1. Married 2. Widowed 3. Separated |  |
| 103 | Religion | 1. Orthodox 2. Muslim 3. Protestant 4. Catholic 5. If other specify______ |  |
| 104 | Ethnicity | 1. Gamo  2. Gofa  3. Amara  4. Wolaita  5. If other specify______ |  |
| 105 | Occupation | 1. Housewife 2. Governmental Employee 3. Merchant 4. Daily Labor |  |
| 106 | Educational Status | 1. Unable to read and write 2. No formal education but can read and write 3. Primary education (1-8) 4. Secondary education (9-12) 5. College or University |  |
| 107 | MUAC | _______________cm |  |

***^*multiple answers are possible^***

Part II: Antenatal related factors

| S/N | Factor | Response | Remark |
| --- | --- | --- | --- |
| 200 | Gravidity | ____ |  |
| 201 | Parity | ____ |  |
| 202 | Birth spacing (Years) | ____ |  |
| 203 | History of adverse pregnancy outcome? | 1. No 2. Yes 3. No |  |
| 204 | ^*^If yes, which one? | 1. Intrauterine fetal death/Stillbirth  2. Preterm  3. Neonatal death  4. Other______ |  |
| 205 | Did you visit a health facility for ANC during your pregnancy for this neonate? | 1. Yes 2. No | If 'No' skip to 203 |
| 206 | If yes, how many times did you receive antenatal care during your time of pregnancy for this neonate? | ________ |  |
| 207 | Where was your ANC visit? | 1. Governmental health center  2. Governmental hospital  3. Privet health institution |  |
| 208 | Did the mother suffer from any of these conditions which are medically confirmed during pregnancy? | 1. Yes 2. No | If 'No' skip to 205 |
| 209 | ^*^ If yes what was the type of complication | 1. Preeclampsia/eclampsia 2. Antepartum hemorrhage 3. Anemia 4. Gestational diabetes   5. DM  6. HTN.  7. Pregnancy-induced HTN  8. Malaria  9. Syphilis  10. Other________ |  |
| 210 | Ever used substances during pregnancy | 1. Yes 2. No | If 'No' skip to 207 |
| 211 | * If Yes, type of substance ever used during pregnancy | 1. Alcohol  2. Khat  3. Cigarette  4.” Gaya”  5. Other |  |
| 212 | Do you have a history of abortion | 1. Yes 2. No |  |
| 213 | If yes, how many times? | 1. One abortion 2. Two abortions 3. Three abortions 4. Four and more abortions |  |

***^*multiple answers are possible^***

Annex VII: Extraction checklist

This checklist is prepared for the collection of intrapartum and neonatal-related information that is important to identify determinants of birth asphyxia among newborns delivered in public hospitals of Gamo and Gofa zones. All this information was retrieved from the client’s medical record without mentioning the name of the client. The information was collected by trained data collectors.

Part I: Intrapartum related factors

| **S/N** | **Factor** | **Response** | **Remark** |
| --- | --- | --- | --- |
| 300 | Was Partograph used | 1. Yes 2. No |  |
| 301 | Presentation | 1. Vertex 2. Breech 3. Face 4. Brow |  |
| 302 | How was the labor started | 1. Spontaneous 2. Induced |  |
| 303 | Labor duration | 1. Normal 2. Prolonged 3. Precipitated |  |
| 304 | Time of membrane rupture | 1. PROM 2. Intrapartum |  |
| 305 | Maternal Fever | 1. Yes 2. No |  |
| 306 | Duration of ROM (hours) | _____ |  |
| 307 | Color of amniotic fluid | 1. Meconium stained 2. Clear |  |
| 308 | Delivery time | 1. Night 2. Day |  |
| 309 | Mode of delivery | 1. SVD 2. C/S 3. Instrumental Delivery |  |
| 310 | If cesarean, anesthesia received by mother during CS was? | 1. General anesthesia  2. Spinal anesthesia |  |
| 311 | Profession of labor attendant | 1. Doctor 2. Midwife 3. Nurse 4. Other(Specify) 5. IESO 6. Other---------- |  |
| 312 | Did the mother suffer from any of the conditions which are diagnosed medically during labor/pregnancy? |  |  |
| 313 | Obstructed labor | 1. Yes 2. No |  |
|  | Maternal hypotension | 1. Yes 2. No |  |
|  | Placenta abruption | 1. Yes 2. No |  |
|  | Placenta Previa | 1. Yes 2. No |  |
|  | HIV | 1. Yes 2. No |  |
|  | Did the neonate suffer from any of the conditions given below? |  |  |
| 314 | Cord Prolapse | 1. Yes  2. No |  |
| 315 | Fetal heart rate | 1. <100, >180 2. 100-180 |  |
| 316 | Oligohydramnios | 1. Yes 2. No |  |
| 317 | Polyhydramnios | 1. Yes 2. No |  |

**Part II: neonatal related factors.**

| **S/N** | **Factor** | **Response** | **Remark** |
| --- | --- | --- | --- |
| 400 | Sex of neonate | 1. Male 2. Female |  |
| 401 | Birth outcome | 1. Singleton 2. Twin 3. Triple and above |  |
| 402 | Birth weight (grams) | _____ |  |
| 403 | Gestational age at birth (weeks) | _____ |  |
| 404 | Birth weight for Gestational age at birth | 1. Appropriate for gestational age 2. Large for gestational age 3. Small for gestational age |  |
